# The secondary prevention of stroke according to cytochrome P450 2C19 genotype in patients with acute large-artery atherosclerosis stroke

**DOI:** 10.18632/oncotarget.24877

**Published:** 2018-04-03

**Authors:** Xingyang Yi, Jing Lin, Ju Zhou, Yanfeng Wang, Ruyue Huang, Chun Wang

**Affiliations:** ^1^ Department of Neurology, People’s Hospital of Deyang City, Deyang 618000, Sichuan, China; ^2^ Department of Neurology, The Third Affiliated Hospital of Wenzhou Medical University, Wenzhou 325200, Zhejiang, China

**Keywords:** clopidogrel, aspirin, ischemic stroke, CYP2C19 polymorphism, outcomes

## Abstract

**Purpose:**

To investigated the effectiveness of antiplatelet agents for the secondary prevention of stroke according to *CYP2C19* genotype in patients with ischemic stroke (IS).

**Methods:**

Between August 2009 and December 2011, 570 acute IS patients with acute large-artery atherosclerosis were randomly assigned to receive either combined clopidogrel and aspirin for the first 30 day, and clopidogrel thereafter (clopidogrel group, n=284) or aspirin monotherapy (aspirin group, n=286). *CYP2C19* genotypes were measured and masked until the end-of-study. The primary outcome was a composite of IS, transient ischemic attack (TIA), myocardial infarction (MI), and death.

**Results:**

During the 5 years follow-up, the primary outcome occurred in 105 patients (18.4%) (71 had IS, 10 had TIA, 12 had MI, and 12 died). There were no significant differences in the primary outcome between clopidogrel group and aspirin group (16.5% vs. 20.3%) or between carriers of the *CYP2C19* reduced-function alleles and noncarriers (21.8% vs.15.7%). In patients with aspirin therapy, *CYP2C19* polymorphism was not associated with the primary outcome. However, in patients treated with clopidogrel, carriers of at least one *CYP2C19* reduced-function allele had a 3-fold higher adjusted risk for primary outcome compared with noncarriers (95% confidence interval, 1.23 to 8.74).

**Conclusions:**

Among IS patients treated with clopidogrel, carriers of a reduced-function *CYP2C19* allele had a significantly higher rate of adverse vascular events than did noncarriers. It should avoid prescribing clopidogrel to these patients with known *CYP2C19* polymorphisms.

## INTRODUCTION

Stroke is one of the leading causes of death among elderly [1]. Patients with ischemic stroke (IS) are at a high risk of developing a recurrent ischemic stroke [2]. Platelet activation plays a key role in the pathogenesis of IS. In IS patients with non-cardiac origin, antiplatelets drugs can decrease the risk of stroke by 11-15%, and the composite risk of stroke, vascular death, and myocardial infarction (MI) is 15-22% [3]. Therefore, antiplatelet therapy, such as clopidogrel or aspirin is recommended for these patients [4]. However, the response to antiplatelet therapy with clopidogrel or aspirin is variable. These findings are suggestive of the existence of clopidogrel or aspirin resistance, i.e., poor or no response to aspirin or clopidogrel treatment. Aspirin resistance was defined as a mean platelet aggregation of ≥20% with 0.5 mg/ml arachidonic acid (AA) and / or a mean aggregation of ≥70% with 10 μM adenosine diphosphate (ADP) by light transmittance aggregometry (LTA) after aspirin intake for 7-10 days [5]. Clopidogrel resistance was defined as < 10% reduction in ADP-induced platelet aggregation from pre-treatment levels, as assessed at 7 -10 days after clopidogrel treatment [6]. Our previous studies showed the prevalences of aspirin and clopidogrel resistance were 25% and 36%, respectively, and antiplatelet drug resistance is associated with a high risk of recurrent ischemic stroke [7, 8].

The mechanisms leading to antiplatelet drug resistance have not yet been fully elucidated and are most likely multifactorial. For example, patient-compliance, absorption or metabolic dysfunction, drug interactions, diabetes, and hypertension may influence the response to antiplatelet drug [7, 8]. However, the genetic etiology of antiplatelet drug resistance has been proposed. There is growing evidence showing that antiplatelet drug sensitivity may be influenced by pharmacokinetic or pharmacogenetic variables, such as metabolism of antiplatelet drug, activation and aggregation of platelet, all of which are further affected by gene single nucleotide polymorphisms (SNPs) [9, 10]. Clopidogrel is an oral thienopyridine-class antiplatelet drug, and has been shown to be superior to aspirin in reducing the risk of ischemic stroke, MI, vascular death in patients with stroke, myocardial infarction, and peripheral vascular disease [11]. It is an inactive prodrug that requires biotransformation to an active metabolite by cytochrome P450 (CYP) enzymes [9]. The expression and function of CYP enzymes definitely have a pivotal role and are associated with the efficiency of clopidogrel. Previous studies have shown that genes that encode CYP enzymes are polymorphic, and that certain alleles confer to reduce enzymatic activities and affect the conversion of clopidogrel into its active metabolite [12]. Our previous studies have demonstrated that carriers of *CYP2C19* loss-of-function (LOF) alleles are associated with clopidogrel resistance, greater risk of stroke and composite vascular events than noncarriers among patients with IS or transient ischemic attack (TIA) treated with clopidogrel [9, 13]. CYP enzymes also metabolize AA into 20-hydroxyeicosatetraenoic acid and epoxyeicosatrienoic acids. These CYP metabolites may cause endothelial dysfunction and platelet activation by a variety of mechanisms [14]. Individual genetic variants of key enzymes in these metabolic processes have been implicated as risk factors of IS. Our previous studies have indicated that variants of CYP genes are not only associated with clopidogrel resistance, but also associated with higher risk for IS in Chinese Population [15]. Thus, for these patients with *CYP2C19* polymorphisms, modification in antiplatelet therapy was reasonable.

Despite antiplatelet drug resistance signifying a risk factor for adverse events, there are no widely accepted treatment recommendations for these patients. The CHANCE (Clopidogrel in High-Risk Patients with Acute Nondisabling Cerebrovascular Events) trial has shown that the combination of clopidogrel and aspirin for the first 21 days is superior to aspirin alone for reducing the risk of stroke in the first 90 days in patients with minor stroke or TIA (8.2% in combination of clopidogrel and aspirin group vs. 11.7% in aspirin group, hazard ratio, 0.68; 95% confidence interval, 0.57 to 0.81; *P* < 0.001) [16]. Our previous randomized-controlled trial has also shown that dual antiplatelet therapy with aspirin and clopidogrel for the first 30 days in acute IS patients was superior to aspirin for reducing ischemic stroke recurrence and neurologic deterioration within 30 days [17, 18]. However, our previous studies and the CHANCE trial had a short follow-up period, the relationship between stroke recurrence after IS and *CYP2C19* genotype status is not well defined.

Several studies have shown that carriers of a *CYP2C19* LOF are associated with efficacy of clopidogrel among IS patients [9, 12, 13]. However, there were no randomized-controlled trials according to *CYP2C19* genotypes to investigate efficacy of clopidogrel and aspirin for the secondary prevention of stroke. The aim of present study was to investigate the effectiveness of antiplatelet agents for the secondary prevention of stroke according to *CYP2C19* genotypes in IS patients on the basis of our previous randomized-controlled trial of dual versus monoantiplatelet therapy in patients with acute large-artery atherosclerosis (LAA) stroke [18]. It may be useful to guide the precise treatment of antiplatelet drugs after IS.

## RESULTS

### Characteristics of the patients

There were no significant differences in demographic or baseline clinicopathological features between the clopidogrel group and aspirin group. Furthermore, there was also no significant difference in incidence of carotid stenosis (stenosis ≥ 50%) between the two groups (17.8% [51/286] in the aspirin group vs. 18.7% [53/284] in the clopidogrel group, *P* = 0.82). The detailed information of the patients was shown in the Table 1 of our previous article [18].

**Table 1:** Baseline characteristics of the study patients

	Patients with primary outcome (n =105)	Patients without primary outcome (n = 465)	*P* value
Age (years)	72.9 ±12.8	66.2 ± 14.8	<0.001
Men (n, %)	61 (58.1)	252 (54.2)	0.48
Body mass index (kg/m^2^)	24.2 ± 3.2	23.9 ± 4.2	0.42
Current smoking (n, %)	51 (48.6)	177 (38.1)	0.047
Hypertension (n, %)	85 (80.9)	329 (70.7)	0.032
Diabetes (n, %)	50 (47.6)	165 (35.5)	0.021
Previous MI (*n*, %)	2 (1.9)	5 (1.1)	0.47
Previous stroke (*n*, %)	0	0	-
Previous angina (n, %)	3 (2.9)	10 (2.2)	0.66
NIHSS score at enrollment	11.2 ± 3.6	11.6 ± 4.5	0.49
Hyperlipidemia (*n*, %)	78 (74.3)	330 (71.0)	0.48
Previous treatment (n, %)			
Antihypertensive drugs	60 (57.1)	256 (55.1)	0.69
Hypoglycemic drugs	35 (33.3)	143 (30.8)	0.63
Statins	13 (12.4)	47 (10.1)	0.47
Aspirin	0	0	-
In-hospital treatment (n, %)			
Thrombolysis	2 (1.9)	9 (1.9)	0.99
Antihypertensive drugs	86 (81.9)	375 (80.6)	0.78
Hypoglycemic drugs	51 (48.6)	192 (41.3)	0.19
Statins	103 (98.1)	458 (98.5)	0.79
Aspirin	58 (55.2)	228 (49.0)	0.26
Clopidogrel plus aspirin	47 (44.8)	237 (51.0)	0.26
*CYP2C19* (rs4244285)			
GG	49 (46.7)	264 (56.8)	0.061
AG +AA	56 (53.3)	201 (43.2)	0.061

MI, myocardial infarction; NIHSS, National Institutes of Health Stroke Scale.

### *CYP2C19* rs4244285 genotype

The Hardy-Weinberg equilibrium test indicated the genotype frequency distribution of *CYP2C19* rs4244285 did not deviate from the Hardy-Weinberg equilibrium (*P* > 0.05). Genotype frequencies for *CYP2C19* rs4244285 wildtype (GG), heterozygous (AG), and homozygous (AA) patients were 54.9% (n = 313), 34.7% (n = 198), and 10.4% (n = 59), respectively. In clopidogrel group (n = 284), genotype frequencies for *CYP2C19* rs4244285 GG, AG, and AA were 54.9% (n = 156), 34.9% (n = 99), and 10.2% (n = 29), respectively. In aspirin group (n = 286), genotype frequencies for *CYP2C19* rs4244285 GG, AG, and AA were 54.9% (n = 157), 34.6% (n = 99), and 10.5% (n = 30), respectively. There were no significant differences in the frequencies of the genotypes for the *CYP2C19* rs4244285 between the two groups (*P =* 0.96). Because the number of homozygous mutation carriers was small, homozygous and heterozygous patients were combined for analyses on the basis of whether they possessed at least one allele with significantly reduced function [12, 19].

### Follow-up for clinical outcomes

Among the 570 patients, 15 patients (2.6%) (7 in clopidogrel group and 8 in aspirin group) were lost to follow-up, 12 patients (2.1%) in clopidogrel group and 8 (1.4%) in aspirin group discontinued the study medication before the end of the study, 5 patients (0.9%) in clopidogrel group and 5 (0.9%) in aspirin group underwent carotid stent therapy during the follow-up period. The primary outcome occurred in 105 patients (18.4%) (71 had IS [12.5%], 10 had TIA [1.8%], 12 had MI [2.1%], and 12 died [2.1%]) during the 5 years follow-up. Compared with patients without primary outcome, the patients with primary outcome were older, had a higher prevalence of hypertension, diabetes mellitus, current smoking (Table 1).

The primary outcome occurred in 47 patients (16.5%) (33 had IS [11.6%], 4 had TIA [1.4%], 5 had MI [1.8%], and 5 died [1.8%]) in clopidogrel group, and as compared with 58 patients (20.3%) (38 had IS [13.3%], 6 had TIA [2.1%], 7 had MI [2.4%], and 7 died [2.4%]) in aspirin group (*P* > 0.05, Table 2). Bleeding events occurred in 45 patients (7.9%). The rate of bleeding events was 8.8% in clopidogrel group as compared with 7.0% in aspirin group (*P* = 0.44, Table 2). There were no significant differences in mild, moderate, and severe hemorrhage between the two groups (Table 2).

**Table 2:** Clinical outcomes

Variable	Aspirin group clopidogrel group		*P* value
	(n = 286)	(n = 284)	
Primary outcome (*n*, %)	58 (20.3)	47 (16.5)	0.26
Ischemic stroke (*n*, %)	38 (13.3)	33 (11.6)	0.54
Transient ischemic attack (*n*, %)	6 (2.1)	4 (1.4)	0.46
Myocardial infarction (*n*, %)	7 (2.4)	5 (1.8)	0.47
Death (*n*, %)	7 (2.4)	5 (1.8)	0.47
Any bleeding event (*n*, %)	20 (7.0)	25(8.8)	0.44
GUSTO mild (*n*, %)	12 (4.2)	14 (4.9)	0.67
GUSTO moderate (*n*, %)	5 (1.7)	7 (2.5)	0.55
GUSTO severe (*n*, %)	3 (1.0)	4 (1.4)	0.69
Gastrointestinal bleeding (n, %)	10 (3.5)	9 (3.2)	0.86
Intracerebral hemorrhage (n, %)	2 (0.7)	3 (1.1)	0.66

GUSTO, Global Use of Strategies to Open Occluded Coronary Arteries.

### *CYP2C19* rs4244285 genotype, clopidogrel, and outcomes

**Figure 1:**
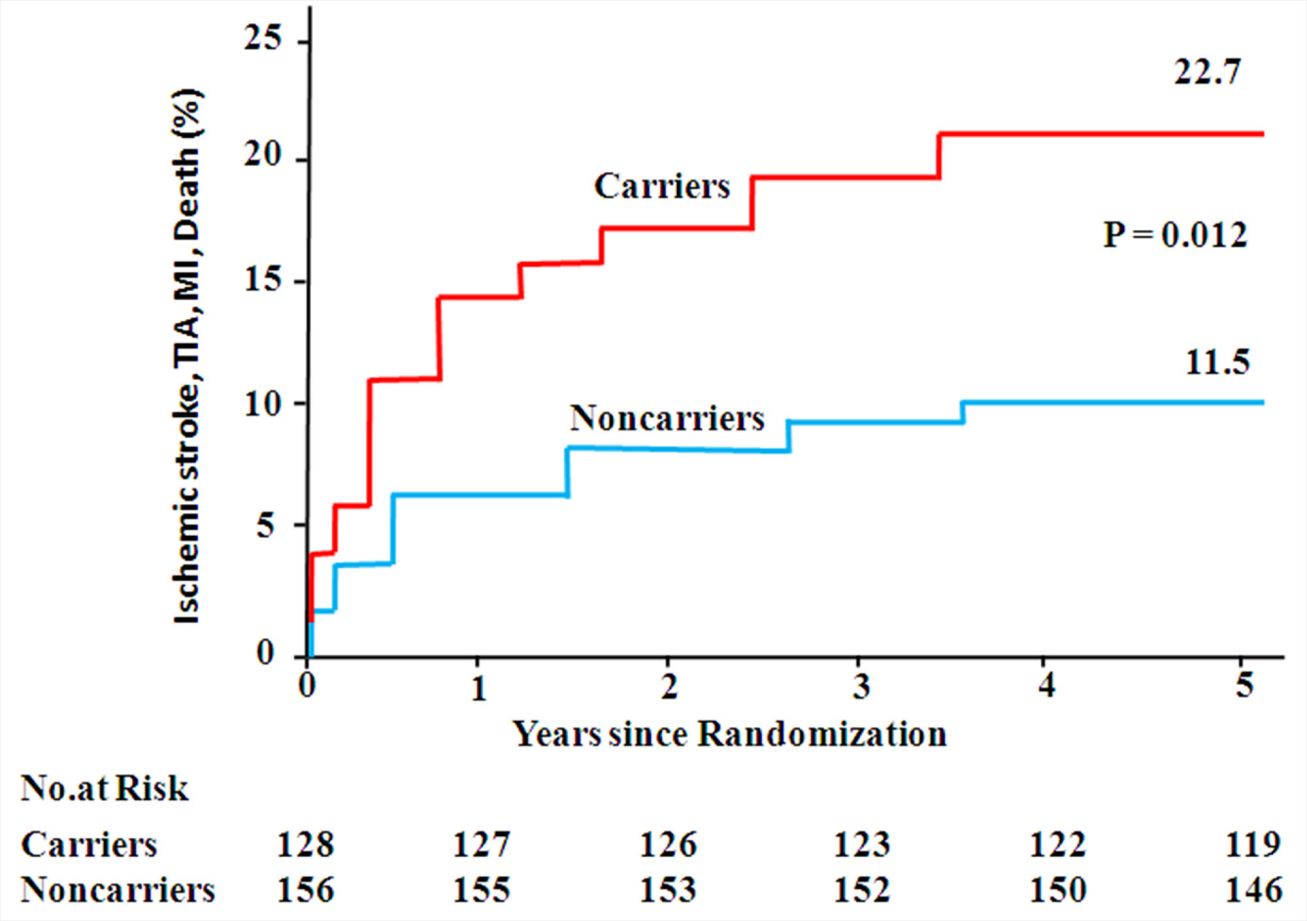
Estimated rates of ischemic stroke, transient ischemic attack, myocardial infarction, or death, according to characteristics of variant-allele polymorphisms In clopidogrel group Figure shows outcomes according to the number of *CYP2C19* loss-of-function alleles. The P values were calculated with the use of multivariate Cox analysis. TIA denotes transient ischemic attack. MI denotes myocardial infarction.

The incidence of primary outcome was 21.8% (56/257) in patients with *CYP2C19* rs4244285 AG/AA (*CYP2C19* reduced-function alleles), as compared with 15.7% (49/313) in patients with *CYP2C19* rs4244285 GG (wildtype genotype) (*P* = 0.061, Table 1). However, among those patients treated with clopidogrel, the patients carrying at least one *CYP2C19* reduced-function allele were at significantly higher risk for the primary outcome of IS, TIA, MI, or death than noncarriers (22.7% [29/128] vs. 11.5% [18/156]; hazard ratio for carriers, 3.02; 95% confidence interval (CI), 1.23 to 8.74; *P* = 0.012) (Figure 1 and Table 3). In patients with aspirin therapy, *CYP2C19* polymorphism did not seem to affect the primary outcome (20.9% [27/129] in the patients carrying at least one *CYP2C19* reduced-function allele vs. 19.7% [31/157] in noncarriers; hazard ratio, 1.25; 95% CI, 0.58 to 2.85; *P* = 0.42) (Table 3).

**Table 3:** Multivariate cox proportional hazards model assessing the risk for primary outcome (Ischemic stroke, TIA, MI, or Death) with respect to the *CYP2C19* polymorphism and use of clopidogrel or aspirin

	HR	95%CI	*P* value
Univariate Models Aspirin users			
Nonarriers of loss-of-function allele	1.0	…	…
Carriers of loss-of-function allele	1.28	0.62 - 2.26	0.34
Clopidogrel users			
Nonarriers of loss-of-function allele	1.0	…	…
Carriers of loss-of-function allele	2.13	1.12 - 5.63	0.001
Model adjusts for age (in quartiles) sex, diabetes, smoking, hyperlipidemia, hypertension, history of myocardial infarction.			
Aspirin users			
Nonarriers of loss-of-function allele	1.0	…	…
Carriers of loss-of-function allele	1.25	0.58 - 2.85	0.42
Clopidogrel users			
Nonarriers of loss-of-function allele	1.0	…	…
Carriers of loss-of-function allele	3.02	1.23 - 8.74	0.012

TIA, transient ischemic attack; MI, myocardial infarction; HR, hazard ratio; CI, confidence interval.

## DISCUSSION

Our previous studies have shown that dual antiplatelet therapy with clopidogrel and aspirin for the first 30 days in patients with acute LAA stroke was superior to aspirin for reducing ischemic stroke recurrence during the first 30 days [18]. In current study, we continued to follow-up the study populations for 5 years, and did not find significant differences in the primary outcome and bleeding events between clopidogrel group and aspirin group. The results were consistent with a subgroup analysis of CAPRIE (Clopidogrel Versus Aspirin in Patients at Risk of Ischemic Events) trial [11]. The CAPRIE trial showed that Long-term administration of clopidogrel to patients with atherosclerotic vascular disease is more effective than aspirin for reducing the combined risk of IS, MI, and vascular death [11]. However, in the subgroup analysis of the stroke patients who entered CAPRIE trial, the effect of clopidogrel was smaller, and did not reach statistical significance (the annual rate of stroke, MI, and vascular death was 7.15% in clopidogrel group vs. 7.71% in aspirin group; 95% CI, 5.7% to 18.7%; *P* = 0.26). The results of CAPRIE trial indicate greater efficacy for clopidogrel as compared with aspirin after symptomatic peripheral vascular diseases than after IS [20].

Key observation in current study was stratified analyses revealed that carriers of at least one *CYP2C19* reduced-function allele had a 3-fold higher risk to have a primary outcome than noncarriers among the patients treated with clopidogrel. These findings indicate that failure of antiplatelet drug therapy with clopidogrel may be related to genetic variations of the *CYP2C19*. Clopidogrel is a prodrug that requires biotransformation to an active metabolite by CYP enzymes. Pharmacodynamic and pharmacokinetic of clopidogrel depend on genetic polymorphism [21, 22]. Carriers of at least one *CYP2C19* reduced-function allele had a reduction of 32% in plasma exposure to the active metabolite of clopidogrel compared with noncarriers (*P* < 0.001) [21]. The loss of function CYP2C19^*^2 genotypes are associated with poor metabolism of clopidogrel, as well as with a higher rate of adverse cardiovascular events in coronary heart disease patients [22, 23]. Similarily, carriers of *CYP2C19* reduced-function alleles are associated with greater risk of composite vascular events than noncarriers in patients with IS treated with clopidogrel [24]. These previous studies and our current study indicate that it is necessary to modify clopidogrel therapy for carriers of *CYP2C19* reduced-function alleles. Our current results indicated that *CYP2C19* polymorphism did not affect the primary outcome in patients with aspirin therapy. The mechanisms of action of aspirin and clopidogrel are different. Aspirin acts by inhibiting cyclooxygenase (COX), thereby preventing thromboxane A2 (TXA2) generation from AA and thrombosis. TXA2 binds to its glycoprotein coupled receptor (GPIIb/IIIa) and platelet membranes receptors (P2Y12, P2Y1) leading to activation of platelet aggregation [25]. COX and platelet receptors or glycoprotein receptor are involved in platelet activation, and are inhibited by aspirin. Although accumulating evidences have suggested that aspirin-relevant genetic variants may affect aspirin responsiveness and clinical adverse outcomes in IS patients [10, 26], the *CYP2C19* is not involved in action of aspirin and thus do not affect aspirin responsiveness and clinical adverse outcomes.

Our previous meta-analysis suggests that carriers of *CYP2C19* loss-of-function allele may be associated with attenuated response to clopidogrel after IS or TIA [9]. Genetic testing may be considered to personalize antiplatelet therapy, especially in East Asian populations for whom the prevalence of *CYP2C19* loss-of-function allele is high [27]. Carriers of *CYP2C19* loss-of-function allele account for ≈30% of white population and up to 50% to 60% in Asians [27–30]. Currently, there are no standardized recommendations of antiplatelet therapy modification for these patients. Increasing the dose of clopidogrel to 225 mg/d might maintain “normal” platelet inhibition response for the patients with *CYP2C19* reduced-function alleles [31]. Dual antiplatelet therapy with aspirin and clopidogrel can more efficiently inhibit platelet activity [17, 18]. However, long-term combination of clopidogrel and aspirin or increasing the dosage of clopidogrel are at higher risk of bleeding complications [32]. Substitution of clopidogrel with another antiplatelet drug (like prasugrel, ticagrelor, cilostazol, or aspirin) is thought to optimize regime, and may help prevent the occurrence of vascular events [33, 34]. A meta-analysis demonstrated that cilostazol was more efficient than other antiplatelet agents for prevention of recurrent stroke in Asian patients [34]. Aspirin prohibits platelet aggregation by inhibiting COX [35]. Our results showed that *CYP2C19* polymorphism did not affect primary outcome in patients with aspirin therapy. Thus, aspirin may be adequate for those patients carrying *CYP2C19* reduced-function alleles.

Our study has several strengths. First, this study was a stratified analysis to investigate the effectiveness of antiplatelet agents for the secondary prevention of stroke according to *CYP2C19* genotypes on the basis of our previous randomized-controlled trial [18]. Second, the follow-up was long for 5 years after acute IS. Third, our current findings could be useful to guide the precise treatment of antiplatelet drugs, decrease the risk of recurrent ischemic events, improve functional status, and develop more effective drugs to prevent recurrent ischemic events after IS. The findings of the current study will lead further research to better understand the research question of interest.

Several important limitations of our study should be considered. First, the plasma clopidogrel levels and its active metabolite were not measured in this study. Therefore, we did not know whether the *CYP2C19* polymorphisms have an effect on plasma clopidogrel levels and its active metabolite. Second, sample size in this study was based on our previous original trial, which were calculated according to the primary outcome of neurological deterioration. This could affect the power of the present analysis. Although the results of present study have shown that carriers of at least one *CYP2C19* reduced-function allele had a 3-fold higher risk for primary outcome compared with noncarriers in patients treated with clopidogrel, the results may not represent the full spectrum of the Chinese population due to the limited sample size and two-center study. Thus, our findings must be validated in larger samples and multi-center studies. Third, we genotyped multiple known functional variants in *CYP2C19* in this cohort of patients, other genetic variants may affact the response to clopidogrel and vascular events [36]. However, some other functional variants were not measured in this study; thus, we could not exclude their role in regulation of the clopidogrel resistance. Finally, only LAA stroke were included in the study. Our findings may not apply to patients with other etiologies stroke who were excluded from this study.

## MATERIALS AND METHODS

### Study populations

This study was conducted in the People’s Hospital of Deyang City and the Third Affiliated Hospital of Wenzhou Medical University. The study protocol was approved by the Ethics Committee of above two participating hospitals. All participants provided written informed consent before participating in this study.

The detailed procedures for the recruitment of IS patients, inclusion criteria and exclusion criteria were described in our previous articles [18]. Briefly, between August 2009 and December 2011, 570 patients who had suffered their first stroke, were due to LAA according to the Trial of ORG 10172 in the Acute Stroke Treatment classification system [37], and were admitted to the above two hospitals within 48 hours of index stroke. All patients were randomly assigned to one of the two treatment groups: aspirin plus clopidogrel (200 mg aspirin and 75 mg clopidogrel for 30 days, and 75 mg/d clopidogrel thereafter, n=284) (clopidogrel group) or aspirin monotherapy (200 mg/d for 30 days and 100 mg/d thereafter, n=286) (aspirin group). Data on various risk factors, including age, sex, body mass index (BMI), hypertension, diabetes mellitus, current smoking, triglycerides (TG), total plasma cholesterol (TC), and low-density lipoprotein cholesterol (LDL-C) were collected.

### Assessment of clinical outcomes

In current study, follow-up was continually performed 3, 6, and 12 months after hospital discharge and thereafter annually by telephone interview, or at the outpatient ward of our department for 5 years. Scheduled follow-up telephone calls were made routinely after discharge to encourage compliance, answer any queries, and record complaints of any side effects.

The primary outcome of this study was a composite of IS, MI, TIA and death. IS was defined as a new focal neurologic deficit lasting for at least 24 hours after onset, diffusion weighted imaging-positive lesion (s) which corresponded to their clinical symptom (s), and proven to be non-hemorrhagic. MI was defined as the presence of at least two of below criteria: prolonged angina > 30 min; electrocardiographic evidence of infarction; total creatinine kinase isoenzyme elevation more than twice the upper limit of normal. TIA was defined as loss of neurological function without residual deficit at 24 hours, and no DWI-positive lesion (s). Death was defined as all-cause mortality.

The safety outcomes were bleeding events. Bleeding events were defined according to the Global Use of Strategies to Open Occluded Coronary Arteries (GUSTO) bleeding classification [38]. Any intracranial hemorrhage or bleeding that causes hemodynamic compromise requiring intervention was defined as GUSTO Severe or life-threatening bleeding. Any bleeding that required blood transfusion in absence of hemodynamic compromise was considered GUSTO moderate bleeding. Any bleeding that did not meet criteria of severe or moderate bleeding GUSTO was defined as mild bleeding.

For those patients who reached at least one of the clinical outcomes, a medical chart review was initiated to determine whether the event met the definitions described earlier. *CYP2C19* genotypes were masked until the end-of-study.

### Genotyping

According to our previous studies [9, 13], the *CYP2C19* rs4244285 single nucleotide polymorphisms (SNPs) were associated with antiplatelet agents resistance and adverse clinical outcomes in acute IS patients. Furthermore, the main aim of the present study was to investigate the effectiveness of antiplatelet agents for the secondary prevention of stroke according to *CYP2C19* genotypes. Thus, we selected and measured *CYP2C19* rs4244285 SNPs in this study.

The genotyping of the SNPs was performed in genomic DNA extracted from periphery blood samples using the matrix-assisted laser desorption/ionization time of flight mass spectrometry method [13]. Each allele of the *CYP2C19* was classified by its known effect on enzymatic function according to literature and with the use of established common-consensus star allele nomenclature [12, 19]. Subjects were dichotomized a priori into two groups on the basis of whether they possessed at least one allele with significantly reduced function.

### Statistical analysis

Differences of characteristics of the patients between clopidogrel and aspirin group, or patients with and without primary outcome were analyzed by univariate methods. Categorical variables are presented as frequencies and percentages and compared using the Chi-square or Fisher’s exact tests. Continuous variables are expressed as mean ± Standard Deviation and compared using the Student’s *t*-test. The Chi-square test was used to assess the deviation of Hardy–Weinberg equilibrium for genotypes. For analyses of the *CYP2C19* genotype in clopidogrel users and nonusers, we considered carriers vs. noncarriers of the mutant alleles (homozygous and heterozygous versus wild-type patients). Because of small numbers within the subgroups, we did not separate analysis for mutated, heterozygous, and wild-type clopidogrel users and nonusers.

Rate of the primary outcome according to patients’genotype and use or nonuse of clopidogrel are showed as a Kaplan-Meier curve, and compared using log rank test. Cox proportional hazards model was used to assess the effect of *CYP2C19* genotypes in clopidogrel users and nonusers on primary outcome. The possible predictor variables into the model to adjust for confounding effects if they: (1) were imbalanced between noncarriers and carriers of the *CYP2C19* polymorphism who were or were not treated with clopidogrel indicated by *P* < 0.2; (2) were established risk factors for stroke between the patients with and without primary outcome. Results of the Cox proportional hazards model are reported as the hazard ratio with the 95% confidence interval (CI). All statistical analyses were performed using SPSS 16.0 (SPSS Inc., Chicago, IL, USA), and all tests were two-sided. A *P* value < 0.05 was considered statistically significant.

## CONCLUSION

Clopidogrel response variability exists in patients with IS. Sequence alterations of *CYP2C19* gene represent one possible mechanism for clopidogrel failure. The genetic variant of *CYP2C19* is associated with higher risk of ischemic vascular events in IS patients receiving clopidogrel therapy. It should avoid prescribing clopidogrel to these patients. Whether the *CYP2C19* polymorphisms could serve as a risk stratification marker to predict possible failure of clopidogrel therapy, and to identify patients who are candidates for alternate antiplatelet agents or higher doses of clopidogrel should be the subject of future testing.

## Abbreviations

IS: ischemic stroke
TIA: transient ischemic attack
MI: myocardial infarction
AA: arachidonic acid
ADP: adenosine diphosphate
LTA: light transmittance aggregometry
LAA: large-artery atherosclerosis
CI: confidence interval
TC: total cholesterol
TG: triglycerides
LDL-C: low-density lipoprotein cholesterol
BMI: body mass index
GUSTO: Global Use of Strategies to Open Occluded Coronary Arteries
SNPs: single nucleotide polymorphisms
NIHSS: National Institutes of Health Stroke Scale
